# Faculty of Prehospital Care, Royal College of Surgeons Edinburgh guidance for medical provision for wilderness medicine

**DOI:** 10.1186/s13728-015-0041-x

**Published:** 2015-12-01

**Authors:** Adrian Mellor, Naomi Dodds, Raj Joshi, John Hall, Sundeep Dhillon, Sarah Hollis, Pete Davis, David Hillebrandt, Eva Howard, Matthew Wilkes, Burjor Langdana, David Lee, Nigel Hinson, Thomas Harcourt Williams, Joe Rowles, Harvey Pynn

**Affiliations:** Academic Department of Military Anaesthesia and Critical Care, RCDM, Birmingham, UK; Cardiothoracic Anaesthesia, James Cook University Hospital, Middlesbrough, TS4 3BW UK; Carnegie Institute for Sport and Human Performance, Leeds Beckett University, Leeds, UK; Academic Critical Care Foundation Doctor, Aberdeen Royal Infirmary, Aberdeen, UK; Centre for Health and Human Performance, London, UK; Summerfield Urgent Care Centre, Birmingham, UK; Department of Emergency Care, University of Birmingham, Birmingham, UK; Faculty of Pre Hospital Care, Royal College of Surgeons of Edinburgh, Edinburgh, UK; The Centre for Altitude Space and Extreme Environment Medicine (CASE Medicine), Institute for Sport, Exercise and Health (ISEH), London, UK; Medical Cell, The Royal Geographical Society, 1 Kensington Gore, London, UK; Primary Care and Occupational Medicine, Ministry of Defence, London, UK; Ultimate Travel Company, London, UK; Department of Emergency Medicine, Defence Medical Services, Whittington, UK; Department of Emergency Medicine, Queen Elizabeth University Hospital and Emergency Medical Retrieval Service, Glasgow, UK; UIAA Medcom, Manchester, UK; British Mountaineering Council, Manchester, UK; Queen Elizabeth Hospital, Birmingham, UK; Adventure Medic Ltd, Edinburgh, UK; Royal Infirmary of Edinburgh, Edinburgh, UK; Adventure Medic, Edinburgh, UK; Expedition and Wilderness Medicine, Devon, UK; Gloucestershire, UK; Department of Emergency Medicine, University Hospitals Bristol, Bristol, UK; Great Western Air Ambulance, Bristol, UK; Wilderness Medical Training, Kendal, UK

**Keywords:** Expedition, Risk assessment, Medical planning, Wilderness medicine, Austere environment

## Abstract

To support leaders and those involved in providing medical care on expeditions in wilderness environments, the Faculty of Pre-Hospital Care (FPHC) of The Royal College of Surgeons of Edinburgh convened an expert panel of leading healthcare professionals and expedition providers. The aims of this panel were to: (1) provide guidance to ensure the best possible medical care for patients within the geographical, logistical and human factor constraints of an expedition environment. (2) Give aspiring and established expedition medics a ‘benchmark’ of skills they should meet. (3) Facilitate expedition organisers in selecting the most appropriate medical cover and provider for their planned activity. A system of medical planning is suggested to enable expedition leaders to identify the potential medical risks and their mitigation. It was recognised that the scope of practice for wilderness medicine covers elements of primary healthcare, pre-hospital emergency medicine and preventative medicine. Some unique competencies were also identified. Further to this, the panel recommends the use of a matrix and advisory expedition medic competencies relating to the remoteness and medical threat of the expedition. This advice is aimed at all levels of expedition medic, leader and organiser who may be responsible for delivering or managing the delivery of remote medical care for participants. The expedition medic should be someone equipped with the appropriate medical competencies, scope of practice and capabilities in the expedition environment and need not necessarily be a qualified doctor. *In addition to providing guidance regarding the clinical competencies required of the expedition medic*, the document provides generic guidance and signposting to the more pertinent aspects of the role of expedition medic.

**Electronic supplementary material:**

The online version of this article (doi:10.1186/s13728-015-0041-x) contains supplementary material, which is available to authorized users.

## Background

The Oxford English dictionary defines an expedition as “a journey undertaken by a group of people with a particular purpose”. This definition highlights the broad scope of expeditions and de facto, expedition medical planning. Medical care provided in an austere environment is often referred to as “wilderness medicine”. This was described by Backer and was defined by its remoteness, physiology, need for improvisation and dependence upon clinical examination and judgement [1]. The scope of this guidance is intended to cover the planning and competencies that facilitate the understanding of the challenges described by Backer and therefore the delivery of good quality clinical care.

The practice of wilderness medicine occurs in many environments and this document is not intended to provide specific advice to specialist expeditions (e.g. deep cave exploration or pioneering extreme new routes in the mountains). The concept of competencies in pre-hospital care has previously been described [2] and *competent individuals are those deemed* to have the “ability to apply knowledge, understanding and skills” to perform to an accepted standard. The competencies discussed consider pre-hospital and primary care skills relevant to medical providers on expeditions in remote areas with some consideration of more specialist environments.

Death and serious injury or illness on expeditions is thankfully rare. Aside from extreme sports in the wilderness, the risks faced by participants on a well-planned expedition are equivalent to those faced by an active person living in the UK. For example, road traffic accidents cause approximately 50 % of unexpected deaths on expeditions per annum [3]. Anderson and Johnson [4] reviewed the data from 246 expeditions with 1263 medical problems (gastrointestinal disease 30 %, medical problems 21 %, orthopaedic problems 19 %, environmental problems 14 %) and a 10 % evacuation rate. More recent published data reviewed charity expeditions over a 5-year period provided by one company. Overall 1564 incidents were reported during 42,482 expedition days. 94 % of the incidents reported were minor and 1 % severe giving a risk of a severe injury or condition of 0.47 per 1000 participant days [5]. Even on potentially high threat expeditions to Denali in Alaska, medical incidents were rare with only 3.5 % of 24,079 climbers requesting medical assistance and only 15 % of these requiring evacuation by the National Park Service [6]. It is worth bearing such figures in mind when planning an expedition, and considering the relatively low prevalence of problems, whilst being mindful of the potentially higher impact should they occur. In addition to medical provision the expedition medic will be responsible for the dental health of participants as well as environmental health. Dental problems, in particular, present a potential burden to the expedition with one expedition reporting 50/309 (16.5 %) of expedition members suffering dental symptoms potentially treatable with a simple dental first aid kit [7].

This document not only provides guidance on the clinical competencies required of the expedition medic but also on other pertinent aspects of the role such as medical planning, risk management, human factors, clinical governance and medical kits.

## Methods

An initial meeting was convened by the FPHC. Members were invited based on their contribution to wilderness medicine in terms of research, teaching, military experience or were selected as representatives of UK-based expedition providers. It was identified that the competencies required for wilderness medicine were wide ranging and evidence for what skills and interventions are required was lacking. For this reason, the panel elected to use the existing FPHC competency framework and adapted it (based on expert opinion) for wilderness medicine use. Members of the panel were then selected to undertake literature reviews and to author specific parts of this consensus document.

The key drivers to any medical plan are:

1. The degree of remoteness of the potential incident.
2. The medical threat—the likelihood of a medical incident occurring.

Remoteness was considered as the time taken to *access* advanced medical care defined in varying ways depending on the injury or illness. For the purposes of this document, it is a facility where a doctor, basic diagnostics, pharmacy, etc., are available and the injury or illness can be managed in a timely and definitive manner. It is accepted that this definition is flexible, as definitive care could potentially be delivered within a well-equipped and appropriately staffed expedition setup and is dependent on the presenting condition.

For the purposes of discussing the required medical competencies, three measures of remoteness from advanced medical care were considered:

1. Time 1: less than 4 h away.
2. Time 2: 4–12 h away.
3. Time 3: more than 12 h away.

These timelines were considered alongside the levels of medical threat that take into account the demographics of the group, the location and the planned activity.

1. Low—such as young, fit group trekking in foothills of Atlas Mountains, Morocco.
2. Medium—such as vehicle borne overland expedition across Eastern Africa with diverse middle aged group.
3. High—such as a ski mountaineering in remote area of Greenland or a medically unscreened group doing charity trek up Mt Kilimanjaro.

Using this model, two main assumptions were made, firsty that time is based on typical estimated travelling time, e.g. summer rather than winter and not worse case. However, planning should take into account a range of travel time most likely to be encountered. Secondly, specific competencies will be dictated by environment (cold, high, hot or any unusual endemic diseases identified by the medical plan).

Priorities for care and evacuation, and therefore competencies for each, could then be agreed upon and are summarised as;

- Less than 4 h: emergency field care.
- 4–12 h: commence definitive treatment in the field.
- 12 h plus: prolonged field care.

**Fig. 1:**
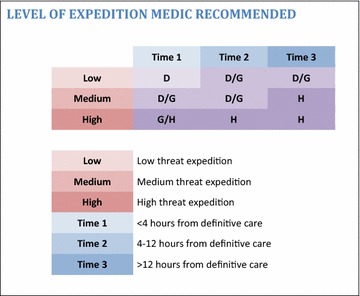
Matrix to determine level of expedition medic required based on remoteness and medical threat of expedition

A suggested level of expedition medic could then be made considering the medical threat and remoteness (Fig. 1). Levels of healthcare provider have previously been established by the FPHC [8].

In summary, these equate to:

- Level D—a non-health care professional with a nationally recognised first aid certificate, caring for patients as a secondary role (such as an expedition leader).
- Level G—a registered healthcare professional working in the expedition environment (such as a junior doctor, nurse or paramedic).
- Level H—an advanced wilderness medicine practitioner (such as a senior doctor with expedition experience).

Once the level of expedition medic has been decided, the competency framework at “Appendix 1” should be used in conjunction with an expedition risk assessment. The competency framework covers most types of expedition—clearly, if the proposed itinerary does not include altitude or diving exposure then those specific competencies will not be required of the expedition medic.

Furthermore, it was recognised by the panel that additional personal skills and attributes may influence who is selected to be the expedition medic. Some of these are discussed within this document.

## Medical planning

The expedition medical plan depends on a fundamental understanding of the risks which are specific to each expedition. Risk assessments are often based on personal experience (or lack of it) and anecdote.

Iserson [9] identified 10 key stages in planning for an extended expedition in a remote location;

1. Optimise workers’ fitness.
2. Anticipate treatable problems.
3. Stock appropriate medications.
4. Provide appropriate equipment.
5. Provide adequate logistical support.
6. Provide adequate medical communications.
7. Know the environmental limitations on patient access and evacuation.
8. Use qualified providers.
9. Arrange knowledgeable and timely consultations.
10. Establish and distribute rationale administrative rules.

An additional key planning stage not included in this original list is knowledge of the planned destination and prevention of illness and problems associated with this area, e.g. malaria, snake envenomation.

All this should be put in place before an expedition leaves to mitigate risk. However, there has to be an acceptance that the provision of medical care in a remote location is inherently challenging and likely to be lacking if measured against what would be available in a developed world healthcare setting.

Understanding the expedition population’s medical needs is fundamental. The support for an expedition of extremely fit experienced mountaineers will be different to that for a charitable trek following similar terrain. Published data can inform estimates of the frequency of likely illnesses, such as altitude illness [4, 10, 11]. Medication and equipment scales can then be decided upon. Providing adequate equipment for unlikely events but with serious consequences is more difficult. Unfortunately, the reality of medicine in remote areas is that severe illness and injury is often non-survivable. In Snowdonia, North Wales, a retrospective data set of 1100 cases brought to the emergency department concluded “there is little or no scope to save any additional lives from trauma in the mountains of Snowdonia” [11].

Communications, logistical support and evacuation routes are all crucial to medical planning. These factors need to be considered along with the nature of the activity to decide on the medical skills required of the provider. With the improvements in global communications and ability to send images, worldwide expert support for management of conditions such as frostbite can be accessed from remote locations. Such links should be established and tested before an expedition leaves as part of the medical plan where possible.

Consideration should be given to medical plans in the absence of the lead expedition medic, i.e. small groups operating from one base location or climbers split across different camps. Diagnostic algorithms for likely conditions such as heat illness or altitude sickness can be placed with medical kits as well as protocols for administration of emergency medication. The lead expedition medic will often be able to communicate emergency medical advice over radio or satellite phone to remote teams, however, algorithms should be robust enough for independent use in emergent situations. The role of expedition medic will include briefing these teams in usage of emergency medical treatments. There is no suggested guidance on the ratio of medics to participants required on an expedition but should be considered on a case-by-case basis in the planning phase.

Medical planning relies on the ability to assess the likelihood of adverse medical events. This is dependent on published data to detect the underlying rate of injury such as discussed above. It is therefore important that, wherever practicable, the incidence of medical problems during expeditions are well recorded and accessible. This is now facilitated by a range of open access journals or online resources.

The purpose of this guideline is to inform best practice and inform expedition planning. It does not seek to provide a mandated framework beyond which none should go. It is accepted that the degree to which the guidelines are implemented may legitimately vary with the nature of the expedition.

## Clinical governance in wilderness medicine

Clinical governance is the framework used to maintain and improve standards of medical care, in which ‘organisations are accountable for continuously improving the quality of their services and safeguarding high standards of care…’ [12].

There are several domains to clinical governance that all have a part to play in an expedition setting:

- Risk management.
- Continuing professional development.
- Evidence based and effective clinical care.
- Audit.
- Patient satisfaction.

These features remain applicable during the pre-expedition, expedition, and post-expedition phases and should not be viewed as optional simply because a practitioner is working outside the health system of the UK. Participants in an expedition should have care provided by someone working within an appropriate scope of practice.

Responsibility for clinical governance rests with both the expedition medic and the expedition organisers. For instance, the organisation must ensure that it carefully selects the expedition medic, that it provides them with timely and accurate information about the participants and the nature of the expedition and that it encourages a culture of openness through the sharing of [medical] risk assessments and post-expedition [medical] reports. The expedition medic is responsible for maintaining their own personal and medical competencies, for precise and robust documentation and for the safe usage and maintenance of medical kit and equipment. Both are responsible for reporting identified problems of any nature and recording these in such a way that incidents can be learned from and mitigated against in the future. Clinical audit should be encouraged.

It is good practice to have a contract between the expedition medic and organisation. An example of such is the UIAA’s Model Contract for Health Care on Trekking and Expeditions [13].

Other factors that the expedition medic and expedition organisers should agree on are listed:

- Provision of medical kit and supply/resupply.
- Work place and distant supervision of expedition medics.
- Responsibility for arranging the provision of specialist medical advice.
- Security and ownership of confidential medical information.
- Responsibility for development and use of Medical Standard Operating Procedures.
- Standardised medical record keeping.

*The liability for providing adequate medical care for all expedition members ultimately lies with the expedition organisers.* In addition, all Level G and H practitioners should discuss any proposed expedition with their professional indemnifiers.

## Risk management

Pre-emptive risk management is essential for managing safety while on expeditions. An understanding of the terms used in risk management is needed to manage risk appropriately.

A threat is something that can cause harm. This may be harm to an individual, to property or to the expedition itself. For example malaria may constitute a threat to an individual, theft is a risk to property and a hurricane may represent a threat to all three. The result of the threat is the consequence of that occurrence.

Likelihood: This is the chance of a threat occurring. For example, acute mountain sickness (AMS) is a threat to which climbers in Scotland will not be exposed. However, for the Himalayan mountaineer, AMS is a threat to which he or she is vulnerable.

The likelihood multiplied by the consequences gives an index of the threat [14]. The assessment of the threat must take place within the context of the expedition. With this context comes the important concept of residual risk. Residual risk describes the risks that remain despite mitigation attempts. For example, while driving a car, a driver may mitigate the risks of crashing by ensuring the car is roadworthy, not driving at night and not exceeding the speed limit. However, the threat of error by another driver causing an accident is difficult to mitigate. This is known as a residual risk.

Once a threat has been assessed and is deemed to be above the threshold of risk for an expedition steps may be taken to reduce the impact of the threat. There are three main ways to mitigate risk:

1. Remove or diminish the threat.
2. Reduce the exposure to the threat.
3. Take measures to reduce the impact of the threat.

For example, an expedition to the Honduran jungle may consider the threat of envenomation by snakebite. The threat may be diminished by ensuring everyone on the expedition wears boots. The exposure to the threat can be reduced by running a teaching session about the snake habitat and how to avoid coming into contact with snakes. The impact could be reduced by ensuring timely evacuation is available to a facility where appropriate care is available. These measures may change an unacceptable risk into a risk accepted by the expedition.

Risk assessment should be carried out at three levels; generic risk assessment for the activity, a daily risk assessment documented for the activity and local conditions and then dynamic risk assessment during the course of the activity.

Incidents that cause harm should be documented, as should ‘near misses’. This will aid future expeditions in building an evidence base of hazards and mitigation strategies. Expedition providers have a legal responsibility for the safety of both paying clients (under Package Travel Regulations 1992) and staff, including any locally employed *staff* (Employer’s Liability). Thorough risk assessment is key to providing both physical and legal protection for both staff and clients.

## Medical threats and mitigation

Expeditions to remote areas are, by their very nature, complex and normal medical risk assumptions and mitigation may not apply.

The experiential evidence backed up by limited published evidence suggest serious incidents on expeditions are unusual [3–6]. Most medical conditions or injuries seen during expeditions can be managed by a competent expedition medic with basic skills. However, incidents in the wilderness environment are compounded by a number of factors;

- The incident occurs in a different location to the expedition medic.
- The casualty may be travelling alone (e.g. between camps in a jungle or on a mountain).
- The casualty may not have the means, capacity or capability to identify their location.
- The casualty may not have the means, capacity or capability to communicate and request help.
- Bad weather/night/visibility/poor communications may hinder the realisation that someone is missing, that a medical incident has occurred and therefore delay any response.

Good medical screening can reduce, but not eliminate, the medical risks to an expedition and should be an essential part of any medical planning. Consideration should be given to who has access to this medically confidential information and whether a certificate and disclosure from the participants’ medical practitioner may be required. In addition to screening, education as to the likely hazards is a key part of reducing the medical risks on an expedition. It should be borne in mind that participants often fail to disclose key medical information and this only comes to light once the expedition starts. Participants should be medically risk assessed again if new information becomes available.

On many expeditions it may be impossible, impractical or unreasonable (as it would fundamentally change the character of the expedition) to provide the highest level of medical care and participants should be sufficiently well informed to accept this risk. Suitable planning and development of guidelines and protocols for management of likely hazards is an important part of medical planning and may remove the need for a medical professional on an expedition.

## Human factors

Human factors refer to the non-clinical aspects of wilderness medicine. It is important to recognise that the role of the expedition medic goes beyond the simple provision of medical care. They often form part of the leadership team, with all the associated responsibilities that this entails.

In the best case, the expedition medic is an independent experienced professional who puts the health and safety of the participants above the objectives of the expedition. For every trip, the expectations and requirements of the expedition medic, from the participants, expedition leaders and the organisers will be subtly different. On occasions, they may even be a source of conflict.

Therefore, the expedition medic does not merely require appropriate clinical skills to deliver care in a wilderness setting but should have the personal skills to work within a team and the technical skills to be able to live comfortably in that environment. A deficiency in any part of the clinical–personal–technical triad will render the expedition medic less effective.

### Personal skills

Personal/interpersonal skills do not always come naturally yet are a vital part of being a functioning, respected team member. The manner in which one employs these ‘soft’ skills will vary depending on the expedition. For example, interaction with a group of ultra-marathon athletes will differ considerably from an inexperienced charity clientele group. The following areas should be considered:

- Communication skills and self-awareness.
- Teamwork.
- Leadership.
- Decision making.
- Coping with fatigue and stress.

The ability to communicate and interact successfully with a team whilst living alongside them is incredibly important, particularly when fostering therapeutic relationships. The expedition medic must be aware of subtle differences in ‘sense of humour’, the need for compassion even with the trivial and regularly reflect on the need to adapt. Instructions or advice should be clear and unambiguous for those to whom they are directed. The expedition medic will often spend the majority of their time as an equal team colleague and friend. It is important to ensure boundaries are well defined and it is clear to participants when there is a swap to the “medic role”.

Leadership styles vary greatly. The expedition medic should be capable of adapting their leadership skills to the needs and requirements of the group. Clear demarcation of roles, responsibilities and decision-making frameworks should be clarified before departure thus minimising the potential for conflict during times of increased stress. Both expedition leader and medic require clarity of jurisdiction, not only during a medical incident/s, but also in a situation where failure to intervene pre-emptively may result in harm.

Decision making on expedition carries with it far more responsibility than purely arriving at a treatable diagnosis. The decisions made will have consequences varying from temporary cessation of activities to permanent casualty evacuation, with all the associated logistical, financial and emotional implications.

The demands placed on the expedition medic have the potential to exceed any other expedition participant. Expedition medics should be prepared to carry out a full day’s expedition activities and then face the possibility of providing the full range of expedition healthcare, irrespective of the time of day or night, including a complex casualty evacuation. Mental resilience and physical fitness are important, as stressors on expedition are many and varied. They include clinical pressures associated with independent/autonomous decision making, stressors of living in a close-knit community or the difficulties of just living and surviving in uncomfortable surroundings with reduced communication with home.

### Expedition skills

The expedition medic will need a range of skills specific to the expedition objectives. These skills are beyond the scope of this document.

Real-life examples of the impact of personal or expedition skill deficiencies can be found at “Appendix 2”.

## Medical kit

Designing and gathering a fit-for-purpose medical kit is frequently overlooked by expedition planners but it is a multifaceted and time-consuming job. It must be clear whose responsibility it will be to provide and pay for medical kit and it must be checked regularly for acceptable quality, including for damage, stock level and out-of-date contents. Meticulous labelling, organisation of the kit and a contents spreadsheet are of paramount importance.

The expedition medic must have knowledge of the indications and side effects of each medication carried, this will depend on the level of medical provider, but any provider must be competent dispensing or administering those medications and be familiar with the identification and timely treatment of any complications occurring. All expedition medics should have access to reference material in this regard. For example, the British National Formulary (BNF) is available electronically as an App.

Medical kits should be bespoke to the expedition in question. Their composition will vary based on team composition, demographics and number of participants as well as destination and the duration of the trip. Kits should reflect the likely illness and injury patterns of the planned activities and to some extent, the level and skills of the expedition medic. Published surveys suggest that first responder medical kits tend to be well equipped to support trauma but less well equipped for medical emergencies [15]. It should also be remembered that the majority of medical presentations on expeditions are not high-level trauma or medical emergencies and medical kits should reflect this by including medications and equipment for treating simple illness and injuries.

Comprehensive advice on provision of medical kits is beyond the scope of this publication. Broad areas for consideration are listed below.

1. A medical kit should be dictated by the medical plan and wilderness environment.
2. Medications (unlike dressings) cannot be improvised and expeditions need to have adequate supplies of trustworthy medications.
3. Import and export restrictions for medications vary between countries.
4. Medications that have a variety of uses should be taken.
5. Practitioners should be aware of expedition members with drug allergies or on regular medications and be aware of any interactions these may have.
6. Group medical kits should be appropriately and securely stored.
7. Ensure adequate means of diluting and administering drugs are available.
8. Individuals should have a personal first aid kit on their person at all times.
9. If travelling in areas with high incidence of HIV or hepatitis consider carrying sterile needles, etc.

These points are expanded in “Appendix 3”.

## Cardiopulmonary resuscitation in the wilderness environment

The decision whether to attempt resuscitation or not in the event of cardio-respiratory arrest in the wilderness is a complex one and requires a pragmatic and realistic decision-making process. Resuscitation efforts and extrication may take place in hazardous terrain and in extreme meteorological conditions. Additionally, resources may be very limited, and there may be multiple casualties amongst who these resources must be shared. Multiple casualty emergencies may fit the definition criteria for a major incident and appropriate Major Incident Medical Management systems may need to be applied in a wilderness setting to effectively utilise available resources.

In 2012, Paal et al. [16] published a position paper to establish scientifically supported guidelines under which cardiopulmonary resuscitation (CPR) could be terminated during mountain rescue. This guidance was subsequently adopted as a formal recommendation by the International Commission for Alpine Rescue (ICAR/CISA) and it is applicable both to medical and non-medical personnel.

As the same principles apply both to organised rescue in the mountains and to wilderness expeditions in terms of decision-making algorithms. The aim of these guidelines is to reduce unnecessary CPR, diminish risk to expedition members or rescuers, apportion limited human and material resources effectively and to identify special circumstances where extended CPR may be indicated.

These circumstances permit the termination of CPR in a patient with unwitnessed loss of vital signs in the wilderness:

1. No return of spontaneous circulation during 20 min of CPR.

AND

2. No special circumstance (see below) warranting extended CPR.

AND

3. When professional medical support is available, either that no shock is advised by an Automated External Defibrillator (AED) at any time, or that only asystole is observed by electrocardiogram (ECG) monitoring.

Special circumstances are hypothermia, lightning strike and submersion (drowning). With these, prolonged CPR may be associated with a good neurological outcome and functional recovery.

## Conclusion

The role of an expedition medic can fall to either medically qualified professionals or to others providing medical care in addition to their primary duty. It is important to recognise that the role of expedition medic is multi-faceted and requires an extensive skill set in addition to suitable underpinning medical knowledge and skills. Expedition medical planning should enable all these aspects to be considered so that appropriate personnel are selected and medical threats recognised and mitigated against.

## Appendix 1

The FPHC competencies are available as an additional file please see Additional file 1.

## Appendix 2

This appendix includes examples of where the expedition medic without the appropriate personal or expedition skills could potentially put themselves and others at risk. These examples are based on the real-life experiences of those on the panel.

1. The expedition medic has never been to altitude and therefore has a lack of environmental experience. As a result is unable to cope with working at altitude and is less effective in providing medical care. Eventually falls prey to altitude illness and has to be evacuated to definitive medical care. The group is left without the originally intended medical care.
2. Expedition medic is required to independently arrive at a casualty in a remote environment. The expedition medic is not competent in navigating and fails to arrive at the casualty. The expedition medic potentially becomes a lost person and requires additional resources to mount a search and rescue effort.
3. Expedition medic lacks situational awareness and as a result becomes targeted by assailants at a market place in a foreign country. They are attacked and robbed of possessions. The expedition medic is psychologically affected for the duration of the expedition and is less effective in providing care with potential long-term health implications.
4. Expedition medic does not have experience in camp craft and lacks necessary personal admin skills. The expedition medic is late each morning in properly organising own equipment. As a result is not ready when the rest of group is ready and either the expedition is delayed or the group is left without the intended medical care until later.
5. A commercial television production taking expedition naive individuals to a hostile environment and filming the outcome. Production aims are to stress individuals physically, socially and mentally whilst filming results. Production company staff have limited understanding of both risk and consequence of harm in the expedition environment and as such encourage risky activities. Intervention by the expedition medic to mitigate risk is frowned upon as this reduces ‘story potential’. These issues will be predicted by experienced expedition medics and mitigated for.
6. Expedition medic is required to treat a casualty on more technical terrain. Expedition medic does not have sufficient technical skills such as appropriate rope work to move competently over technical terrain. They become stranded as a result and require rescuing.
7. A production company wish to film a sequence where a presenter is attempting to recover a vehicle trapped in soft sand. Expedition porters are placing rocks and sand ladders in front of spinning wheels whilst the presenter is positioned behind the vehicle at great risk of being hit by flying debris. An astute and experienced medic with identify a significant risk of injury to the presenter and intervene promptly.

The above examples can happen to anyone even with sound non-medical skills and experience in the wilderness environment. However, expedition medics that have the required operational capability reduce any risk.

## Appendix 3

This annex composes some of the lessons identified from the experience of the panel with regard to preparing an expedition medical kit.

1. Know your environment and adapt the team medical kit accordingly. For example, for tropical environments where the risk of infection is high, take broad spectrum antibiotics, a malarial detection kit (with high sensitivity) and stand-by treatment. For high altitude environments, include medications following the most recent guidance in the treatment of acute mountain sickness, high altitude pulmonary and cerebral oedema.
2. You cannot improvise medications. Dressings and splints can be improvised whereas medications cannot be. You cannot guarantee the quality of medications bought in many countries so whilst they may be easily available, they may not be as efficacious.
3. Know the import and export restrictions for countries. Know the Medicines Health and Regulatory Agency (MHRA) scheduling of different drugs and the restrictions that this imposes. Be aware of the restrictions imposed by other countries; for example, drugs such as codeine are robustly regulated in the Middle Eastern countries. The FCO website is a useful resource for more details of restrictions for individual countries.
4. Take medications with more than one use. For example, codeine has analgesic, antitussive and anti diarrhoeal properties so is extremely versatile. Antibiotics such as co-amoxiclav and azithromycin have broad spectrums of cover so can be used to treat a wide range of infection.
5. Beware of interactions between medicines in the medical kit. For example, ciprofloxacin and ibuprofen in combination can reduce the seizure threshold so make epileptics more prone to seize. Be aware what regular medications are being taken by group members and compile the group medical kit accordingly.
6. Choose the most appropriate container for the medical kit. Be aware that in a tropical environment, the medical kit will need to be stored in a damp proof, sealable container.
7. Ensure that all participants have their own personal medical kits containing basic medical supplies such as blister prevention and treatment, simple analgesia, dressings and a plentiful supply of their own regular medication.
8. Be aware that certain medications used for intramuscular injection have specific diluents. For example, ceftriaxone for intramuscular injection uses 1 % lignocaine for reconstitution and injection. This is particularly important for groups where the medic is not confident or unable to achieve intravenous cannulation.
9. If travelling to regions of the world with a high incidence of HIV, consider taking a set of sterile needles and cannulae in the event that a participant requires local hospital admission.
10. Remember that other issues not normally associated with developed world medicine will fall to the expedition medic. For example, issues with contact lenses and hearing aids. Contact lenses can be problematic on expedition. The risk of keratitis is greater in contact lens wearers. Ensure all participants that plan to wear contact lenses take their glasses in addition. Ensure that anyone with a hearing aid takes spare batteries and that you and they know how to change them. If participants travel with specific pieces of equipment to manage their condition, consider asking for a demonstration on usage before the trip, for example, an insulin pump. A plan for dealing with failure of equipment (e.g. insulin pump) should be in place.

## Additional file

10.1186/s13728-015-0041-x Expedition medic competencies.

## Abbreviations

AED: automated external defibrillator
AMS: acute mountain sickness
BNF: British National Formulary
CPR: cardiopulmonary resuscitation
ECG: electrocardiogram
FPHC: Faculty of prehospital care of the Royal College of Surgeons of Edinburgh
ICAR: International Commission for Alpine Rescue
UIAA: International Climbing and Mountaineering Federation (Union International des Associations d’Alpinisme)
