# Supplementary material for: Faculty of Prehospital Care, Royal College of Surgeons Edinburgh guidance for medical provision for wilderness medicine
Source: Extrem Physiol Med. 2015 Dec 1;4:22. doi: 10.1186/s13728-015-0041-x (PMC4665843; doi:10.1186/s13728-015-0041-x)
Supplement: Supplementary file 1 — 10.1186/s13728-015-0041-x Expedition medic competencies. [file 13728_2015_41_MOESM1_ESM.pdf]

THE ROYAL COLLEGE OF SURGEONS OF EDINBURGH

EXPEDITION MEDIC COMPETENCIES

The competencies in green in the primary care section are specific to Level D practitioners only

		D	G	H
**PRIMARY CARE**				
Cardiovascular				
General	Be able to recognise classical ischaemic heart pain, TIAs and CVAs, provide immediate treatment and make rational decisions on need and mode of evacuation	x		
Assessment	Be able to assess and interpret cardiovascular symptoms and signs and differentiate common ailments		x	x
Angina	Recognise and treat angina and be able to make rational decisions on the need for, and mode of, evacuation		x	x
Acute coronary syndromes	Recognise acute cardiac events including myocardial infarction and dysrhythmias (SVT, AF etc), treat where possible, and be able to make rational decisions on the need for, and mode of, evacuation		x	x
DVT	Be able to differentiate, with some confidence, DVT from other lower leg pain		x	x
TIA and CVA	Be able to confidently diagnose, make rapid assessment (FAST) and make rational decisions on the need for, and mode of, evacuation	x	x	x
Respiratory				
General	Have a good overview of asthma, understand basic treatments and be able to assist in improvising delivery devices; appreciate simple assessments of respiratory rate and shortness of breath and make rational decisions on the need for, and mode of evacuation	x		
Assessment	Be able to assess and interpret respiratory symptoms and signs and differentiate common ailments		x	x
Dyspnoea	Have a good understanding of the differential diagnosis and relevant treatments		x	x
Cough	Be able to determine, with some certainty, the cause of cough and appropriate treatments		x	x
Asthma	Have a comprehensive understanding of the BTS guidelines for both acute and chronic asthma and be able to manage step up and down plans as well as acute events		x	x
Pneumonia	Understand how to assess and manage a case of pneumonia and be able to make rational decisions on the need for, and mode of, evacuation		x	x
Endocrinology				
General	Have a basic understanding of diabetes, be aware of symptoms and signs of hypoglycaemia and hyperglycaemia and be able to provide emergency treatment and appropriate evacuation arrangements for both.	x		
Diabetes	Have a comprehensive understanding of how to modify diabetic treatment during episodes of ill health and in response to altered meal times/exercise/dietary intake, as well as management of diabetic emergencies. Also to be able to advise on managing time zone changes, glucose monitoring in adverse conditions and storage of drugs and testing equipment in different environments		x	x
Gastrointestinal				
General	Be conversant with simple measures for management of diarrhoea and vomiting; have a working knowledge of what constitutes an 'acute abdomen' and an understanding of the importance of prompt evacuation	x		
Assessment of the abdomen	Be able to assess and interpret abdominal symptoms and signs, and differentiate common ailments		x	x
Acute abdomen	Be able to distinguish abdominal emergencies from milder conditions and be able to make rational decisions on the need for, and mode of, evacuation		x	x
Vomiting and diarrhoea	Have a broad understanding of different causes and their respective management plans		x	x
Constipation	Be able to recognise and offer treatment options for constipation		x	x
Dyspepsia and reflux	Be able to offer dietary advice as well as treatment options		x	x
Haemorrhoids	Have a good understanding of the different degrees of pile, how they may be managed and be able to make rational decisions on the need for evacuation.		x	x
Renal and urology				
Assessment of urinary symptoms	Be able to assess and interpret renal/urinary symptoms and signs and differentiate common ailments		x	x
Renal colic and stones	Understand the natural history of renal colic/stones and be able to confidently distinguish it from other abdominal emergencies		x	x
UTIs	Be able to instigate an appropriate management plan		x	x
Assessment of testicular pain	Be able to recognise and treat the different causes of testicular pain and be able to make reasoned decisions on the need, and mode, of evacuation		x	x
Musculoskeletal				
Assessment of musculoskeletal pain	Be able to recognise common causes of musculoskeletal pain and appreciate their impact on function	x	x	x
Fractures and dislocations	Have a good understanding of management of fractures/dislocations, including the ability to make cogent decisions on the need, and mode of, evacuation. Be able to apply standard splintage and have a good working knowledge of the principles of improvised splintage	x	x	x
Low back pain	Be able to separate out those patients with back emergencies and those with benign disease and make appropriate management plans including coherent decisions on need for, and mode of, evacuation	D	x	x
Soft tissue injuries	Have a good understanding of soft tissue injuries relating to exercise and trauma. Be able to discriminate between those problems that are liable to come to harm from further activity and those in whom this is unlikely. Be able to apply simple and common strapping techniques	x	x	x
Neurology				
Assessment	Be able to assess and interpret neurological symptoms and signs and differentiate common ailments		x	x
Epilepsy	Have a good understanding of primary care management of fits	x	x	x
Vasovagal	Be able to confidently distinguish faints from more malignant conditions	D	x	x
Headache and migraine	Be able to assess and provide a differential diagnosis for headache with appropriate management plans		x	x
Dermatology				
Assessment	Be able to assess and interpret dermatological symptoms and signs and differentiate common ailments		x	x
Blisters	Be able to manage blisters	x	x	x
Burns	Be able to recognise severity of burn and manage appropriately	x	x	x
Eczema, fungal, viral and bacterial skin infections	Confidently diagnose and treat simple skin conditions		x	x
Ingrowing toe nail, paronychial infection and subungual haematoma	Be able to undertake minor surgery to treat these conditions		x	x
Infectious disease				
Assessment	Be able to recognise common infectious diseases		x	x
Meningitis	Be conversant with emergency management of meningitis and need for evacuation	x	x	x
Immunology				
Assessment	Be able to assess and interpret infective/reactive symptoms and signs and differentiate common ailments		x	x
Allergy and anaphylaxis	Be able to manage both simple allergic reactions and anaphylaxis	x	x	x
Gynaecology & Sexual Health				
Assessment	Be able to assess and interpret gynaecological and obstetric symptoms and signs and differentiate common ailments		x	x
Ectopic pregnancy	Be able to assess risk and likelihood of ectopic pregnancy and make appropriate evacuation plans as part of management		x	x
Contraception	Understand common methods of contraception and be able to offer advice		x	x
ENT				
General	Be able to confidently manage epistaxis	x		
Assessment	Be able to assess and interpret ENT symptoms and signs and differentiate common ailments		x	x
Sore throat, sinusitis, earache, dizziness and vertigo, motion sickness, epistaxis	Have a comprehensive understanding of common ENT conditions and their management	D	x	x
Quinsy	Be able to recognise and manage appropriately		D	x
Ophthalmology				
Assessment	Be able to assess and interpret eye symptoms and signs, and differentiate common ailments.	D	x	x
Eye trauma	Be able to irrigate an eye, remove superficial foreign bodies and treat minor trauma appropriately.	D	x	x
The red eye	Be able to distinguish between benign and more serious causes of red eye and the treatment of these conditions.			x
Eye emergencies	Have an understanding of what constitutes an eye emergency and how to manage this, including sensible decisions on need for evacuation.	D	x	x
Mental Health				
General	Be able to recognise commonly presenting mental health conditions such as depression and anxiety and be familiar with basic treatment measures	x		
Mental health assessment	Be able to conduct a primary care level mental health assessment		x	x
Anxiety	Understand the aetiology and treatment of anxiety, both chronic and acute		x	x
Depression and deliberate self harm	Understand depression and be able to perform a primary care level self-harm risk assessment	D	x	x
Psychosis	Be able to recognise psychosis and make informed decisions on management including evacuation		x	x
Adjustment	Have a good understanding of adjustment reaction and management options	x	x	x
	Understand the implications of the expedition environment on team members	x	x	x
	Management of psychological reactions to traumatic events	x	x	x
Capacity and consent	Have a basic understanding of capacity and consent	x	x	x
Eating disorders	Be familiar with the presentation and management of common eating disorders	x	x	x
Substance abuse	Understand and be able to recognise common patterns of substance abuse, their immediate management and be able to make cogent decisions on the need for evacuation	x	x	x
Psychology	Appreciate the range of behaviours found within any population, including a broad understanding of Aspergers, Adult ADHD, personality traits/disorders and how individuals with these are best supported in a group situation	x	x	x
**TRAUMA**				
Safety and Scene Management	Have the ability to take on the leadership role at an incident and have a good understanding of the capabilities of other staff/helpers	x	x	x
	Have a good understanding of scene safety	x	x	x
	Be able to practice dynamic risk assessment of the scene	x	x	x
	Be able to perform dynamic risk assessment of casualties taking into account casualty safety	x	x	x
	Be competent in triage sieve, sort & management skills for multiple casualties	D	x	x
	Be able to make decisions on casualty evacuation and appropriate secondary care	D	x	x
Catastrophic bleeding	Recognise life-threatening haemorrhage	x	x	x
	Effectively manage catastrophic limb bleed	x	x	x
	Effectively manage catastrophic junctional bleed	D	x	x
	Able to apply direct pressure	x	x	x
	Able to apply indirect pressure	x	x	x
	Competent application of tourniquet	x	x	x
	Competent in use of haemostatic dressing / packing a wound with gauze		x	x
Spinal injury	Appreciate mode of injury high risk for spinal injury	x	x	x
	Appropriate C-spine management	x	x	x
	Put head in neutral alignment	x	x	x
Airway	Inspect and clear airway	x	x	x
	Head tilt chin lift	x	x	x
	Jaw thrust	x	x	x
	Postural airway management	x	x	x
	Use of suction	D	x	x
	Size and insert nasopharyngeal airway	D	x	x
	Size and Insert oropharyngeal airway	D	x	x
	Size and insert supraglottic airway device		x	x
	Manage choking patient	x	x	x
	Be able to competently determine the need for surgical airway and carry it out			x
Breathing	Identify if patient is breathing normally	x	x	x
	Correctly assess breathing rate, depth and quality	x	x	x
	Perform basic chest examination	x	x	x
	Identify life-threatening chest conditions	x	x	x
**Management of tension pneumothorax**	Recognition of tension pneumothorax development	D	x	x
	Finger thoracostomy			x
	Needle decompression		x	x
	Chest drain insertion			x
**Management of sucking chest wound**	Recognition of a sucking chest wound	D	x	x
	Application and management of occlusive chest dressings	D	x	x
**Management of flail chest**	Recognition of immediately life-threatening flail chest	D	x	x
	Splintage of immediately life-threatening flail chest	D	x	x
	Appropriate pain management to assist ventilation	D	x	x
**Management of massive haemothorax**	Recognition and appropriate management of massive haemothorax/ internal chest bleeding			x
	Understanding of environmental limitations of pulse oximetry		x	x
	Ability to monitor & react to end tidal CO2 if available			x
**Administration of oxygen**	Safely configure an oxygen system for use	D	x	x
	Free-flow oxygen application (with correct mask choice)	D	x	x
	Nebulisation of medications		x	x
	Oxygen delivery via bag-valve-mask		x	x
	Use of expired air ventilation (mouth-to-mouth/to nose)	x	x	x
	Use of pocket-mask ventilation	x	x	x
Circulation	Assess presence of circulation	x	x	x
	Measure pulse rate and rhythm	x	x	x
	Assess blood pressure		x	x
	Measure capillary refill time	x	x	x
	Assessment of heart sounds		x	x
	Assessment of blood loss	x	x	x
**Haemorrhage control (not prev listed)**	Elevation of limb	x	x	x
	Wound closure		x	x
	Splintage as a haemorrhage control method	D	x	x
	Application of pelvic splintage	D	x	x
	Use of traction devices	D	x	x
	Attain intravascular access		x	x
	Administration of appropriate intravenous fluids		x	x
	Application of appropriate wound dressings	x	x	x
Disability	Be able to effectively use the AVPU assessment of conscious level	x	x	x
	Assessment of pupil reaction + size	x	x	x
	Identify indicators of underlying head injury	x	x	x
	Assessment of traumatic brain injury		x	x
	Perform a more in depth neurological examination		x	x
	Assess neurovascular status			x
	Assess blood glucose level	D	x	x
	Demonstrate a knowledge of the pharmacology of analgesic and anaesthetic agents used in remote medicine		D	x
Extended Care	Have a good understanding of positioning of patients during care, paying attention to airway and potential for pressure areas	x	x	x
	Understand the importance of trends in observations and be able to instigate appropriate monitoring with respect to temperature, pulse, blood pressure, respiratory rate, urinary output etc	x	x	x
	Be able to adequately prepare a patient for evacuation, including packaging and administration	x	x	x
Evacuation	Be conversant with improvised methods of extrication	x	x	x
	Have knowledge of international retrieval systems and the methods by which patients can be repatriated from remote world-wide locations	x	x	x
	Possess a theoretical knowledge of the common rescue systems in use by aeromedical operations world-wide (e.g. long-line, winch and their limitations)	x	x	x
**EXPEDITION MEDICINE**				
Environmental health	Understand the importance and provision of basic hygiene and be able to offer pre-event planning advice, implement basic practical preventative measures and counsel participants on the same	x	x	x
	Be able to oversee systems of food preparation that ensure high standards of hygiene	x	x	x
	Have a good understanding of the range of available water disinfection systems and manage the provision of safe drinking water	x	x	x
	Appreciate basic concepts of sanitation and be able to supervise sensible latrine location and construction	x	x	x
	Be able to offer guidance on disposal of 'grey water' and all waste	x	x	x
Travel Medicine	Have knowledge of where to signpost expedition members regarding pre-travel vaccinations and malaria prophylaxis	D	X	X
	Be able to intelligently assess country specific health risk and develop the knowledge and skills to manage any such illnesses/injuries including the preparation of appropriate drugs and equipment		x	x
Tropical	Have the ability to differentiate a fever in the tropics		x	x
	Be able to remove tick / bot fly / tumbu fly / jigger flea / leech	D	x	x
	Recognition of rashes - dengue / cutaneous larva migrans / ECM		D	x
	Application of pressure immobilsation following snake bite	D	x	x
Dentistry	Ability in differential diagnosis and medical management of dental pain	D	x	x
	Ability in differential diagnosis and management of facial pain	D	x	x
	Competent in placement of dental filling/ dressing	D	x	x
	Correctly assess and replant an avulsed front tooth	D	x	x
	Awareness of landmarks and techniques to achieve local dental anaesthesia		D	x
	Awareness of the principles and technique of dental extraction		D	x
	Awareness of risk levels and management of dental infection involving fascial spaces			x
**ENVIRONMENTAL INJURY**				
Drowning	Recognition and management of unconscious drowned patient	x	x	x
	Recognition and management of conscious drowned patient	x	x	x
	Recognition of late complications of drowning		D	x
Heat injury	Have a comprehensive knowledge of heat illness including risk, prevention, recognition and management	x	x	x
	Recognise and manage heat stroke and heat exhaustion	x	x	x
Cold injury	Have a comprehensive knowledge of local cold injury (NFCI, frostbite) and be able to manage appropriately in terms of risk prevention, diagnosis and treatment	x	x	x
	Recognise possibility, prevent and if required treat hypothermia	x	x	x
	Be aware of specialist advisors for cold and heat illness		x	x
High Altitude	Understand "normal" physiology at high altitude	D	x	x
	Understand the impact of high altitude environment on pre-existing disease	D	x	x
	Understand pathophysiology and mitigation of altitude illness	D	x	x
	Recognise signs and symptoms of AMS	x	x	x
	Recognise signs and symptoms of HAPE	x	x	x
	Recognise signs and symptoms of HACE	x	x	x
	Provide simple treatments for AMS, HACE, HAPE	x	x	x
	Use specific medication for AMS, HAPE, HACE (inc IV/IM)	x	x	x
	Appropriate use of medical oxygen	D	x	x
	Understand the use and limitation of climbing oxygen systems	D	x	x
	Appropriate use of a portable hyperbaric chamber	D	x	x
	Demonstrate a knowledge of the pharmacology of the the agents used to manage the high altitude pathologies			x
	Understand specific issues around treatment of avalanche victims	D	x	x
**EXPEDITION PLANNING AND LOGISTICS**				
Characteristics of medical provider	The medical provider should have the capacity to work autonomously and self sufficiently under testing conditions but also have the insight to know when to evacuate and handover care	x	x	x
	They should act selflessly and always put the patient, or potential patient, above their own personal aspirations in the outdoors	x	x	x
	The medical provider must have a standard of fitness comparable to the group, be competent in any required outdoor skills, and have good leadership qualities	x	x	x
Pre-expedition planning	Understand the particular patterns of injury and associated pathology common to wilderness sports and activities (e.g. falls in mountaineering, avalanche incidents in skiing)	x	x	x
	and the associated additional effects from environmental injury (frostbite, hypothermia, hyperthermia etc)			
	Be able to formulate a simple risk assessment matrix for the event and identify any appropriate mitigatory measures. Risk assessment should include country specific and event specific health risk, identification of in country medical facilities, casualty	D	x	x
	Have the requisite knowledge to medically screen potential event participants including those with pre-existing medical conditions; to offer advice on their suitability for inclusion and risk minimisation		x	x
	Have a good understanding of travel medicine, be able to offer basic advice to participants on country specific health risks and signpost them to comprehensive sources of information for risk reduction		x	x
	Be able to compile a medical kit that reflects the demographics of the group and the type and location of the event	D	x	x
Communications	Have a comprehensive working knowledge of all modes of communication available for working in remote areas including mobile and satellite phones, radios and additional e-communication systems. Appreciate the pros and cons of all methods	x	x	x
	Be able to quickly establish effective lines of communication between team members (both at location and remote) in an emergency situation.	x	x	x
	Be practised in relaying information to outside agencies in a recognised and systematic manner	x	x	x
	Have the skills to provide an effective handover of clinical care when transferring patients	D	x	x
**MISCELLANEOUS**				
Death	Understand how accepted resuscitation algorithms must be adapted for the wilderness (e.g. in hypothermic cardiac arrest) and understand the circumstances where conventional CPR is futile and ought not to be attempted	x	x	x
	Be conversant with current guidelines on confirmation of death	D	x	x
	Be able to manage complex logistics surrounding a death	D	x	x
	Be able to offer simple early grief counselling	x	x	x
Medico-legal	Be aware of the complex medico-legal aspects of practicing medicine overseas in remote locations, commercial or otherwise		x	x
